# Multimeric antibodies from antigen-specific human IgM^+^ memory B cells restrict *Plasmodium* parasites

**DOI:** 10.1084/jem.20200942

**Published:** 2021-03-04

**Authors:** Christopher D. Thouvenel, Mary F. Fontana, Jason Netland, Akshay T. Krishnamurty, Kennidy K. Takehara, Yu Chen, Suruchi Singh, Kazutoyo Miura, Gladys J. Keitany, Eric M. Lynch, Silvia Portugal, Marcos C. Miranda, Neil P. King, Justin M. Kollman, Peter D. Crompton, Carole A. Long, Marie Pancera, David J. Rawlings, Marion Pepper

**Affiliations:** 1 Center for Immunity and Immunotherapies, Seattle Children's Research Institute, Seattle, WA; 2 Department of Immunology, University of Washington School of Medicine, Seattle, WA; 3 Vaccine and Infectious Diseases Division, Fred Hutchinson Cancer Research Center, Seattle, WA; 4 Laboratory of Malaria and Vector Research, National Institute of Allergy and Infectious Diseases, National Institutes of Health, Rockville, MD; 5 Department of Biochemistry, University of Washington School of Medicine, Seattle, WA; 6 Malaria Infection Biology and Immunity Section, Laboratory of Immunogenetics, National Institute of Allergy and Infectious Diseases, National Institutes of Health, Rockville, MD; 7 Institute for Protein Design, University of Washington, Seattle, WA; 8 Department of Pediatrics, University of Washington School of Medicine, Seattle, WA

## Abstract

Multimeric immunoglobulin-like molecules arose early in vertebrate evolution, yet the unique contributions of multimeric IgM antibodies to infection control are not well understood. This is partially due to the difficulty of distinguishing low-affinity IgM, secreted rapidly by plasmablasts, from high-affinity antibodies derived from later-arising memory cells. We developed a pipeline to express B cell receptors (BCRs) from *Plasmodium falciparum*–specific IgM^+^ and IgG^+^ human memory B cells (MBCs) as both IgM and IgG molecules. BCRs from both subsets were somatically hypermutated and exhibited comparable monomeric affinity. Crystallization of one IgM^+^ MBC-derived antibody complexed with antigen defined a linear epitope within a conserved *Plasmodium* protein. In its physiological multimeric state, this antibody displayed exponentially higher antigen binding than a clonally identical IgG monomer, and more effectively inhibited *P. falciparum* invasion. Forced multimerization of this IgG significantly improved both antigen binding and parasite restriction, underscoring how avidity can alter antibody function. This work demonstrates the potential of high-avidity IgM in both therapeutics and vaccines.

## Teaser

Thouvenel et al. isolate BCR sequences from *Plasmodium*-specific IgM^+^ human memory B cells, expressing them as both IgM and IgG molecules. Multimerization confers superior antigen binding and parasite restriction, highlighting an important role for memory B cell–derived IgM in immunity.

## Graphical Abstract

**Figure:**
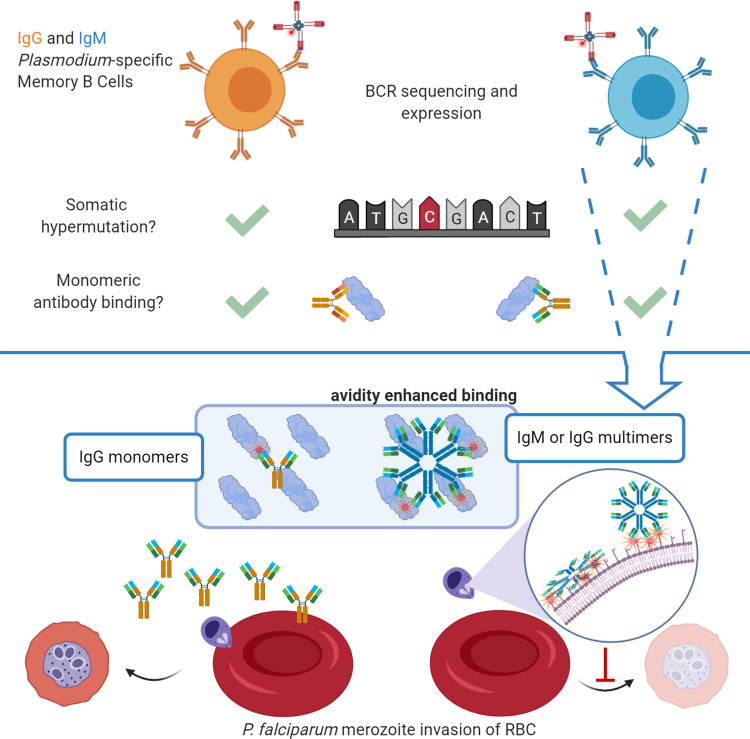

## Introduction

The humoral immune response is characterized by B cell production of antibodies that protect against infection. As the humoral response develops, B cells can adopt diverse fates and secrete different antibody isotypes (Corcoran and Tarlinton, 2016). Naive follicular B cells express surface IgM molecules, and IgM is generally the first antibody isotype to be secreted in response to infection. IgM antibodies have historically been considered to be of lower quality (i.e., lower affinity) than IgG antibodies. This is perhaps due to the two distinct waves of high IgM and IgG serum antibody levels that follow a primary infection or immunization (Eisen, 2014). Early IgM production comes largely from unmutated plasmablasts before class-switching. Although class-switched plasmablasts can contribute to the early antibody response, the production of somatically hypermutated high-affinity IgG from long-lived, germinal center–derived plasma cells dominates the later stages of controlled infection and is associated with immediate protection from subsequent challenge (Victora and Nussenzweig, 2012). Direct comparisons of the functional attributes of IgM and IgG have been difficult to make given the technical challenges of expressing and purifying IgM molecules compared with IgG molecules (Gautam and Loh, 2011; Chromikova et al., 2015). Perhaps consequently, although monoclonal antibodies comprise the largest and fastest-growing segment of the biopharmaceuticals industry (Gautam and Loh, 2011; Ecker et al., 2015; Kesik-Brodacka, 2018), the vast majority of the >570 antibodies currently in development are IgG molecules (Kaplon and Reichert, 2019). Meanwhile, the therapeutic potential of IgM antibodies is only beginning to be explored in depth.

Despite this historical neglect, evidence of the efficacy of IgM antibodies in fighting infection has begun to accumulate. IgM has the unique ability to form pentamers and hexamers, enabling high-avidity interactions with antigen (Brewer et al., 1994). Protective serum IgM antibodies against bacterial, viral, and eukaryotic pathogens have been identified in rodents and humans (Brown et al., 2002; Alugupalli et al., 2003; Harada et al., 2003; Kato et al., 2015; Boyle et al., 2019). An IgM antibody induced by influenza infection in mice was shown to have higher binding capacity than a clonally identical IgG molecule due to increased avidity, and efficiently conferred protection from flu in mice and ferrets (Shen et al., 2019).

Several recent reports have specifically examined the role of IgM in protection from malaria, caused by infection with parasites of the genus *Plasmodium*. Mice selectively deficient in IgM experience greater parasitemia and mortality compared with wild-type mice upon infection with *Plasmodium chabaudi*, a model for uncomplicated *Plasmodium falciparum* infection (Couper et al., 2005). Anti-parasite IgM antibodies arising from natural exposure can activate complement to inhibit growth of *Plasmodium* parasites in vitro and are significantly associated with protection from clinical malaria in human cohorts (Boyle et al., 2019; Kurtovic et al., 2018; Mbengue et al., 2019). Studies of genetically distinct ethnic groups in West Africa, who experience identical parasite exposure but exhibit different susceptibilities to malaria, have found that resistant and susceptible groups display strongly divergent breadth and magnitude of *Plasmodium*-specific IgM responses, whereas IgG responses are far more similar between groups (Bolad et al., 2005; Arama et al., 2015). Consistent with these findings, human volunteers vaccinated with attenuated sporozoites generate somatically hypermutated, high-affinity IgM antibodies to liver-stage antigens (Tan et al., 2018), though it remains unclear whether such IgM antibodies can confer sterile protection in vivo.

These combined observations suggest that parasite-specific IgM antibodies may be an important component of naturally acquired immunity to malaria. Consistent with this notion, IgM-expressing memory B cells (MBCs) have recently been recognized as rapid and potent responders to a secondary *Plasmodium* infection, a finding that challenges the dogma that serum IgM is primarily derived from low-affinity unmutated plasmablasts early in the primary immune response. We and others have identified somatically hypermutated, high-affinity, long-lived *Plasmodium*-specific IgM^+^ MBCs that respond to challenge in mice and humans (Arama et al., 2015; Tan et al., 2018; Krishnamurty et al., 2016; Zenklusen et al., 2018; Kisalu et al., 2018). Consistent with an important role in protection, *P. chabaudi*–specific IgM^+^ MBCs responded more rapidly than IgG^+^ MBCs in a murine challenge model (Krishnamurty et al., 2016).

Taken together, these previous observations raise the unanswered question of whether IgM-expressing MBCs may represent a critical source of affinity-matured, high-avidity antibodies that can participate in protective anamnestic responses. To address this question, we isolated individual *P*. *falciparum*–specific IgM^+^ and IgG^+^ MBCs from humans with a history of repeated malaria. To test the binding and protective capacities of IgM antibodies in their physiological multimeric state, we expressed the same isolated B cell receptor (BCR) sequence as either a monomeric IgG or a multimeric IgM molecule. We found that a somatically hypermutated IgM antibody, derived from an IgM^+^ MBC and specific for a linear epitope within the conserved *Plasmodium* protein merozoite surface protein 1 (MSP1), possessed a multimerization-dependent antigen-binding capability that was significantly higher than a clonally identical monomeric IgG molecule. Further, forced multimerization of the IgG antibody similarly improved its performance over the monomeric form. These findings underscore how multimerization can alter antibody function and support the concept that IgM antibodies derived from MBCs may play an important and underappreciated role in protection from parasites.

## Results

**Figure 1.**
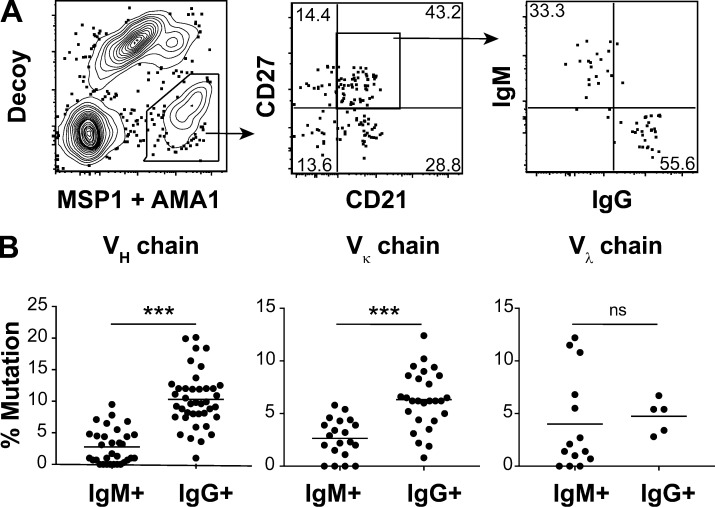
**Isolation of somatically hypermutated, parasite-specific IgM^+^ MBCs from human subjects with repeated *Plasmodium* exposure.** **(A)** Gating strategy to sort single CD21^+^CD27^+^, IgM^+^, or IgG^+^ MBCs specific for the *P. falciparum* antigens MSP1-19 or AMA1 from PBMCs of Malian subjects with a history of repeated malaria (*n* = 9). **(B)** BCRs from eight donors were sequenced and the number of somatic hypermutations per 100 nucleotides was measured in the heavy chains (V_H_) and both light chains (Vκ and Vλ) by comparison to germline sequences. (For IgM and IgG respectively: *n* = 32, 40 for V_H,_ *n* = 20, 27 for Vκ, and *n* = 14, 5 for Vλ.) ***, P < 0.001 by unpaired two-tailed *t* test. ns, not significant.

**Figure S1.**
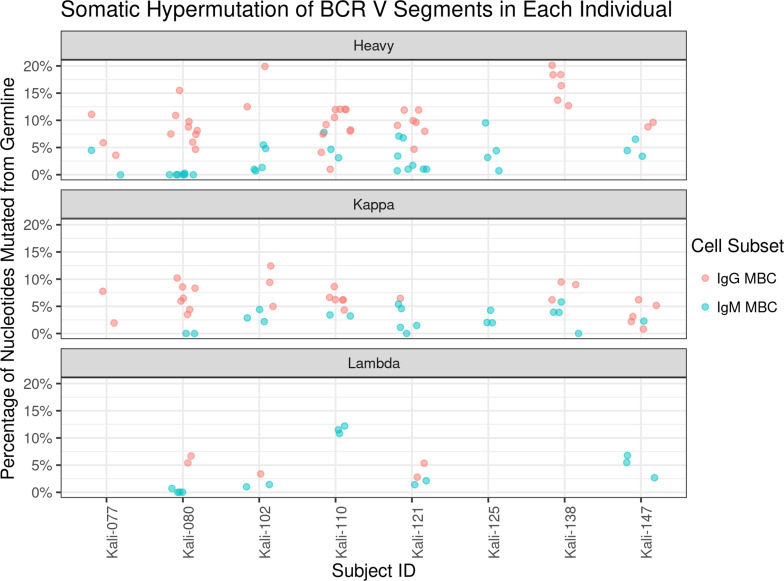
**Somatic hypermutation of BCR V segments in each study donor.** BCR sequences from each of the eight donors, delimited by subject identification (ID), were aligned to the germline immunoglobulin genes and alleles in the IMGT database. The number of somatic hypermutations per 100 nucleotides was measured and is displayed according to chain and derived cell subset.

To study the role of IgM in protection from malaria, we developed a pipeline to isolate and express BCR sequences from *P. falciparum*–specific human IgM^+^ MBCs. First, we used fluorophore-conjugated tetramers (Krishnamurty et al., 2016) to sort single classically defined CD21^+^CD27^+^, IgM^+^, or IgG^+^ MBCs (Tangye and Good, 2007) specific for the blood-stage antigens MSP1 (the 1-19 fragment) and apical membrane antigen 1 (AMA1) from human peripheral blood mononuclear cells (PBMCs). The human donors were 10–15 yr of age and live in a region of Mali with intense malaria transmission (Tran et al., 2013). MSP1 and AMA1 are both surface proteins that facilitate erythrocyte invasion during the blood stage of disease (Beeson et al., 2016). A decoy reagent was used to identify and exclude B cells that bound to parts of the tetramer other than our antigens of interest (Taylor et al., 2012). Although the tetramers incorporated antigens from a laboratory strain of *P. falciparum* (3D7), they readily labeled B cells from humans with natural exposure to diverse *Plasmodium* strains (Fig. 1 A), suggesting epitope conservation. Of the 208 antigen-specific CD21^+^CD27^+^ cells analyzed by flow cytometry, 111 (53%) were IgM^+^. We performed nested PCR on each single cell and obtained at least one heavy or light chain sequence from 93 cells, including 50 IgG^+^ MBCs and 43 IgM^+^ MBCs. We were not able to recover both heavy and light chains from every cell, but included all recovered sequences in further analysis (Table S1). Alignment to germline sequences in the international ImMunoGeneTics information system (IMGT) revealed that the majority of IgM^+^ cells encoded somatically mutated receptors, albeit with lower mutation frequencies than IgG^+^ MBCs (Fig. 1 B and Fig. S1).

**Figure 2.**
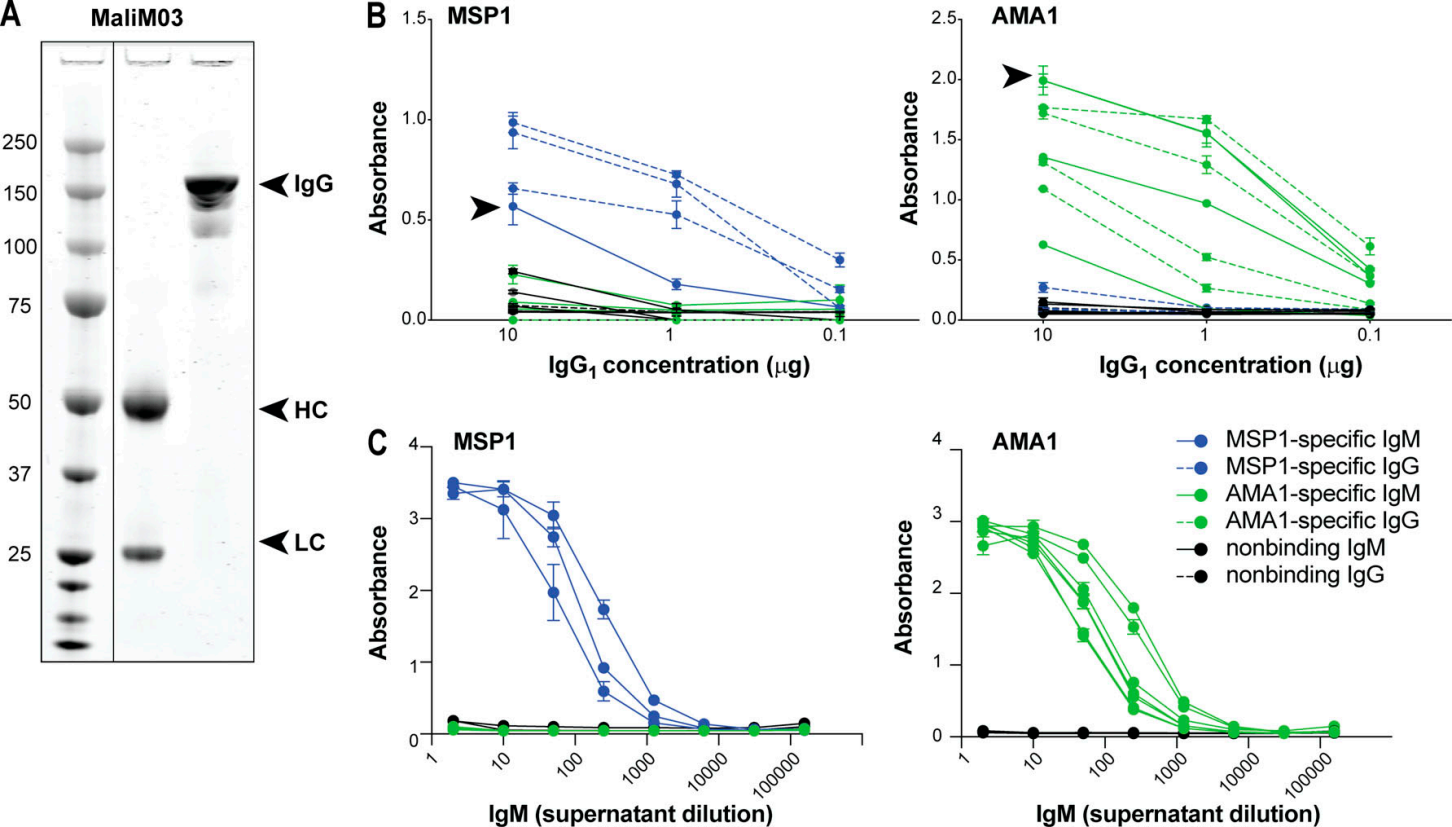
**Antibodies cloned from MSP1- and AMA1-specific IgM^+^ and IgG^+^ MBCs bind target antigens.** **(A)** Western blot showing the IgM^+^ MBC-derived clone MaliM03 expressed as IgG1 under denatured (middle lane) or native (right lane) conditions. Arrows indicate the heavy (HC) and light (LC) chains in the denatured sample, and the assembled IgG molecule in the native sample. **(B)** ELISAs demonstrating the affinity of recombinant IgG1 antibodies derived from either IgG^+^ MBCs (dashed lines) or IgM^+^ MBCs (solid lines) for either *P. falciparum* 3D7 MSP1 (left panel) or AMA1 (right panel). Arrowheads mark the antibodies we named MaliM03 (left graph) and MaliA01 (right graph). **(C)** The IgM^+^ MBC-derived antibodies from B that did not bind AMA1 or MSP1 when expressed as IgG1 molecules were reexpressed as multimeric IgM molecules and retested for antigen-binding by ELISA. Antibodies that were judged positive for binding to MSP1 are shown in blue; those binding to AMA1 are in green. Graphs show mean + SD from one of two independent experiments; within each experiment, each sample was tested in triplicate.

For initial characterization of MBC-derived IgM and IgG antibodies, we cloned paired BCR variable region sequences from 28 individual MBCs sorted from eight donors (*n* = 20 IgM, 8 IgG). All heavy chains were cloned into vectors with a γ1 heavy chain constant region, and light chains were cloned into vectors with their original constant region of either κ or λ. Each heavy chain–light chain pair cloned from the same single cell was expressed as an IgG1 molecule (Fig. 2 A). This approach enabled direct comparison of antibody affinity by removing differences in avidity between IgG and IgM and allowing detection of all antibodies with the same secondary reagent. Antibody binding to AMA1 or MSP1 was confirmed by ELISA (Fig. 2 B). Among antibodies cloned from IgG^+^ MBCs, seven out of eight bound to AMA1 or MSP1, but not to an irrelevant control protein, demonstrating antigen specificity. However, only 4 of the 20 antibodies cloned from IgM^+^ MBCs bound AMA1 or MSP1 when expressed as IgG1 molecules (Fig. 2 B and data not shown). We reasoned that some IgM-derived antibodies might require higher avidity for efficient antigen binding. To examine this possibility, we expressed the 16 nonbinding IgM^+^ MBC-derived antibodies as multimeric IgM molecules and retested them for AMA1 and MSP1 binding by ELISA. One of these exhibited poor expression and was set aside. Of the 15 antibodies that expressed well as IgM molecules, 10 now bound either AMA1 or MSP1 as IgM molecules (Fig. 2 C). Pooling these results with the IgG1 ELISA data, we concluded that of the 28 antibodies cloned from tetramer-labeled MBCs, 21 (7 IgG-derived and 14 IgM-derived) were specific for MSP1 or AMA1. Further screening revealed that three antibodies (one IgG-derived and two IgM-derived) bound both MSP1 and AMA1, albeit with differing binding capacities, which could reflect either antigen cross-reactivity or binding to a common epitope introduced in the recombinant proteins for technical purposes (such as the HIS-tag used for protein purification). These dual-binding antibodies were excluded from further analysis.

Because we were most interested in the highest-quality (i.e., best-binding) antibodies, we returned to those clones that bound antigen when expressed as IgG1 molecules. Among these, the sole MSP1-specific antibody derived from an IgM^+^ MBC, which we named MaliM03, exhibited a binding capacity within the lower range of IgG^+^ MBC-derived antibody binding. Strikingly, an AMA1-specific IgM^+^ MBC-derived antibody, which we named MaliA01, bound at least as well as the highest-binding antibody from an IgG^+^ MBC (Fig. 2 B). The small number of antibodies tested does not permit generalization about the relative affinities of IgM- versus IgG-derived sequences, but our data do suggest that variable regions derived from IgM^+^ MBCs are capable of developing affinities comparable to those of IgG antibodies.

**Figure 3.**
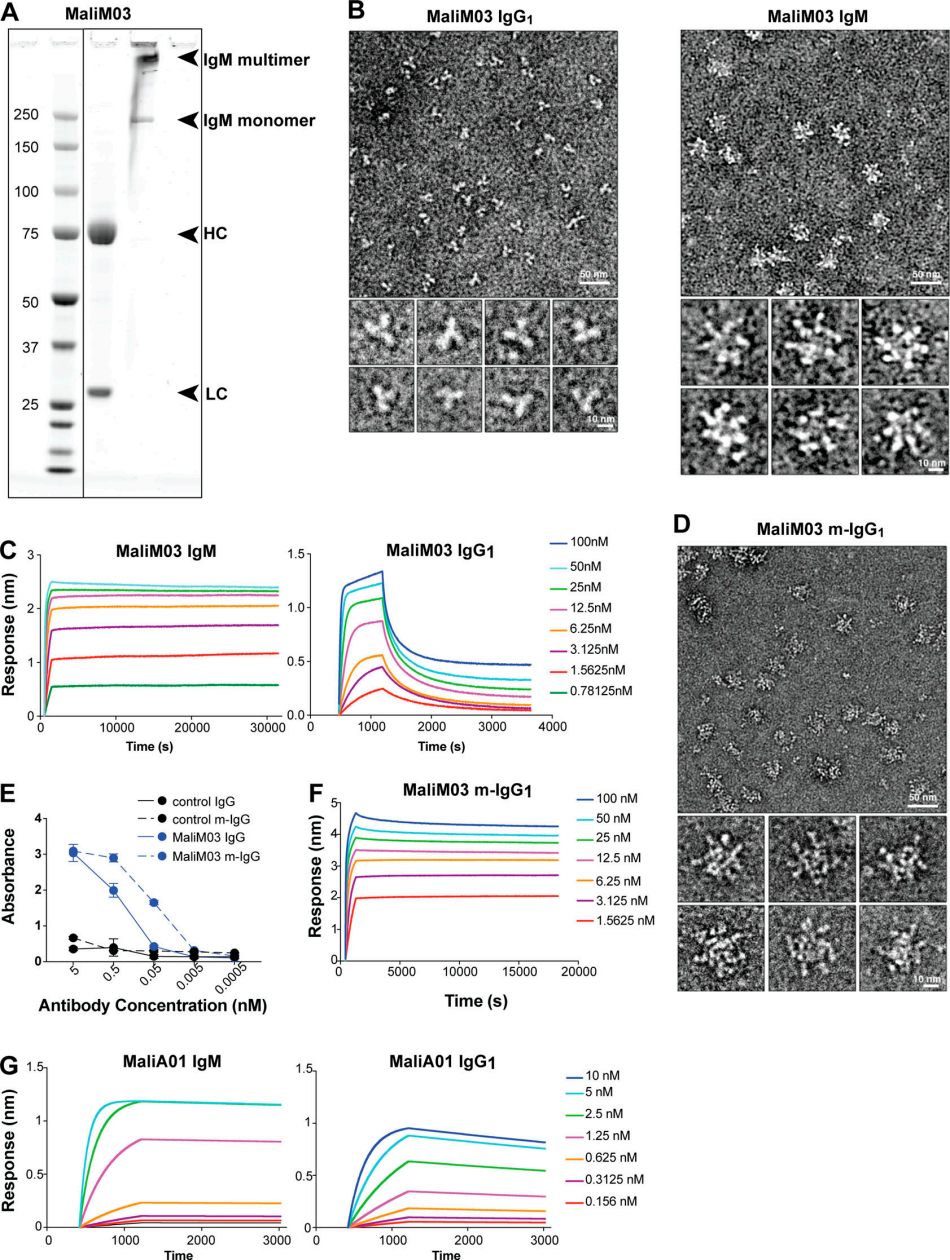
**Multimerization confers exponentially stronger antigen binding to an IgM antibody compared with a clonally identical monomeric IgG antibody.** **(A)** Western blot showing the antibody MaliM03 expressed as a multimeric IgM molecule and analyzed under either denaturing (middle lane) or native (right lane) conditions. Arrows indicate the heavy (HC) and light (LC) chains in the denatured sample, and both multimeric and monomeric bands in the native sample. **(B)** The same antigen-binding region of MaliM03 was expressed either as an IgG1 or an IgM molecule by fusion with the appropriate constant region. The structure of each antibody was assessed by negative-stain electron microscopy. Insets show individual IgG monomers (left) or IgM multimers (right). **(C)** The binding properties of MaliM03 IgG1 (left) and IgM (right) for MSP1-19 were measured by BLI. **(D)** The MaliM03 binding region was fused to a modified IgG constant region that self-assembles into multimers (MaliM03 m-IgG), and the multimeric structure was confirmed by negative-stain electron microscopy. **(E)** Binding of the MaliM03 m-IgG to MSP1 was measured by ELISA and compared with binding of the monomeric IgG antibody, as well as monomeric and multimeric forms of an irrelevant control antibody. **(F)** BLI measurement of binding of MaliM03 m-IgG to MSP1. **(G)**. BLI measurements of MaliA01 binding to AMA1 when expressed as multimeric IgM (left) or monomeric IgG1 (right). Data in C and E–G are representative of two independent experiments. In E, error bars indicate SD.

Dissection of IgM function in host protection has been hampered by the technical challenges of expressing and purifying large multimeric IgM complexes (Gautam and Loh, 2011; Chromikova et al., 2015). To circumvent these difficulties, most past characterizations of IgM antibodies have been performed by expressing the antigen-binding domains of sequenced IgM molecules with a standardized IgG constant region, as we did above. However, this approach precludes examination of the role of the IgM constant region, and specifically of the high-avidity interactions endowed by multimerization. To compare the functional properties of multimeric IgM to monomeric IgG, we expressed the BCR variable regions from the MSP1-specific IgM^+^ MBC clone (MaliM03) fused to the secreted μ constant region, which predominantly self-assembles into >990 kD IgM multimers (Randall et al., 1992). The expressed antibody, obtained from transient transfection of 293T cells, was purified on an IgM affinity column that preserved its multimeric form (Fig. 3 A). We verified the quaternary structure of our recombinant IgM antibody using negative stain electron microscopy. The MaliM03 IgM molecule formed both pentamers and hexamers in solution, whereas an antibody with an identical variable region and an IgG1 constant region (MaliM03 IgG1) was monomeric as expected (Fig. 3 B).

While antibodies derived from both IgG^+^ and IgM^+^ MBCs demonstrated comparable affinities by ELISA when expressed as monomers with an IgG1 constant region, we reasoned that the native, pentameric IgM molecules should exhibit greater antigen-binding capacity than a monomeric IgG with the same binding region, due to increased avidity. Our ELISA screens provided support for this reasoning, with some antibodies testing negative when expressed as IgG monomers but efficiently binding antigen as IgM multimers (Fig. 2, B and C). To more precisely analyze the contributions of multimerization, we used Bio-Layer interferometry (BLI) to measure the antigen-binding capacity of MaliM03 expressed either as an IgM or IgG1 antibody. Strikingly, the MaliM03 IgM antibody exhibited no appreciable dissociation from its cognate antigen over the course of an 8-h assay; in contrast, MaliM03-IgG1 clearly dissociated from the antigen, indicating a significantly weaker apparent affinity (Fig. 3 C). The result with the IgM molecule is consistent with binding of a multimeric complex: individual antigen-binding regions may dissociate from antigen periodically, but the likelihood that all regions dissociate simultaneously is small—akin to a starfish lifting one arm from a surface while its remaining four arms keep it in place. Thus, these two antibodies with identical affinities (stemming from identical binding regions) have vastly different capacities for sustained binding to antigen due to avidity.

To directly test the role of multimerization in increasing the binding capacity of MaliM03, we modified the constant region of the MaliM03 IgG molecule to include an 18–amino acid tail piece from IgM as well as one additional mutation (L309C), elements previously demonstrated to be sufficient to induce multimerization (Sørensen et al., 1996). After confirming by negative stain electron microscopy that the modified IgG antibody assembled into multimers (Fig. 3 D), we measured its binding to antigen. By ELISA, the multimeric IgG (m-IgG) bound MSP1 approximately tenfold better than its monomeric form (Fig. 3 E). To more precisely quantify its binding capacity, we again turned to BLI. Like MaliM03 IgM, and in contrast to the IgG monomer, the m-IgG bound antigen so tightly we could not observe dissociation from antigen, confirming that multimerization dramatically enhances the binding capacity of this antibody (Fig. 3 F). Together, these results raise the possibility that in vivo*,* affinity-matured IgM^+^ MBCs give rise to serum antibodies that may be more effective at binding antigen than IgG antibodies of similar affinity.

To assess the generalizability of our findings with MaliM03, we characterized the antigen-binding properties of another IgM^+^ MBC-derived antibody from our screen, MaliA01, which our ELISA experiments identified as a specific and potent binder of AMA1 (Fig. 2 B). We used BLI to compare the binding capacity of MaliA01 expressed as either an IgG1 or an IgM molecule. As observed for MaliM03, the multimeric MaliA01 IgM molecule bound AMA1 more strongly than its IgG1 counterpart, confirming the effect of avidity on enhancing binding. The difference in binding between the two isotypes was not as dramatic as for MaliM03, however (Fig. 3 G). We hypothesized that the antigen-binding region of MaliA01 may have a stronger affinity for its antigen than MaliM03, as suggested by the binding data obtained using monomeric IgG1 (Fig. 2 B; and Fig. 3, C and G); higher affinity could result in a less critical role for multimerization in strong antigen binding. We performed additional BLI studies to assess binding in the absence of avidity contributions and found that MaliA01 has an affinity (dissociation constant) of 8.59 × 10^−9^ M, which is within observed ranges for mouse monoclonal (IgG) antibodies (Shen et al., 2019; Stubenrauch et al., 2013). However, we were unable to obtain a reliable dissociation constant value for MaliM03, so we could not compare the affinities of the two antibodies directly. Altogether, given that both MaliM03 and MaliA01 were derived from IgM^+^ MBCs, our results suggest that IgM antibodies can achieve strong antigen binding in several ways, via both high affinity of the monomer and enhanced avidity of the multimer.

**Figure 4.**
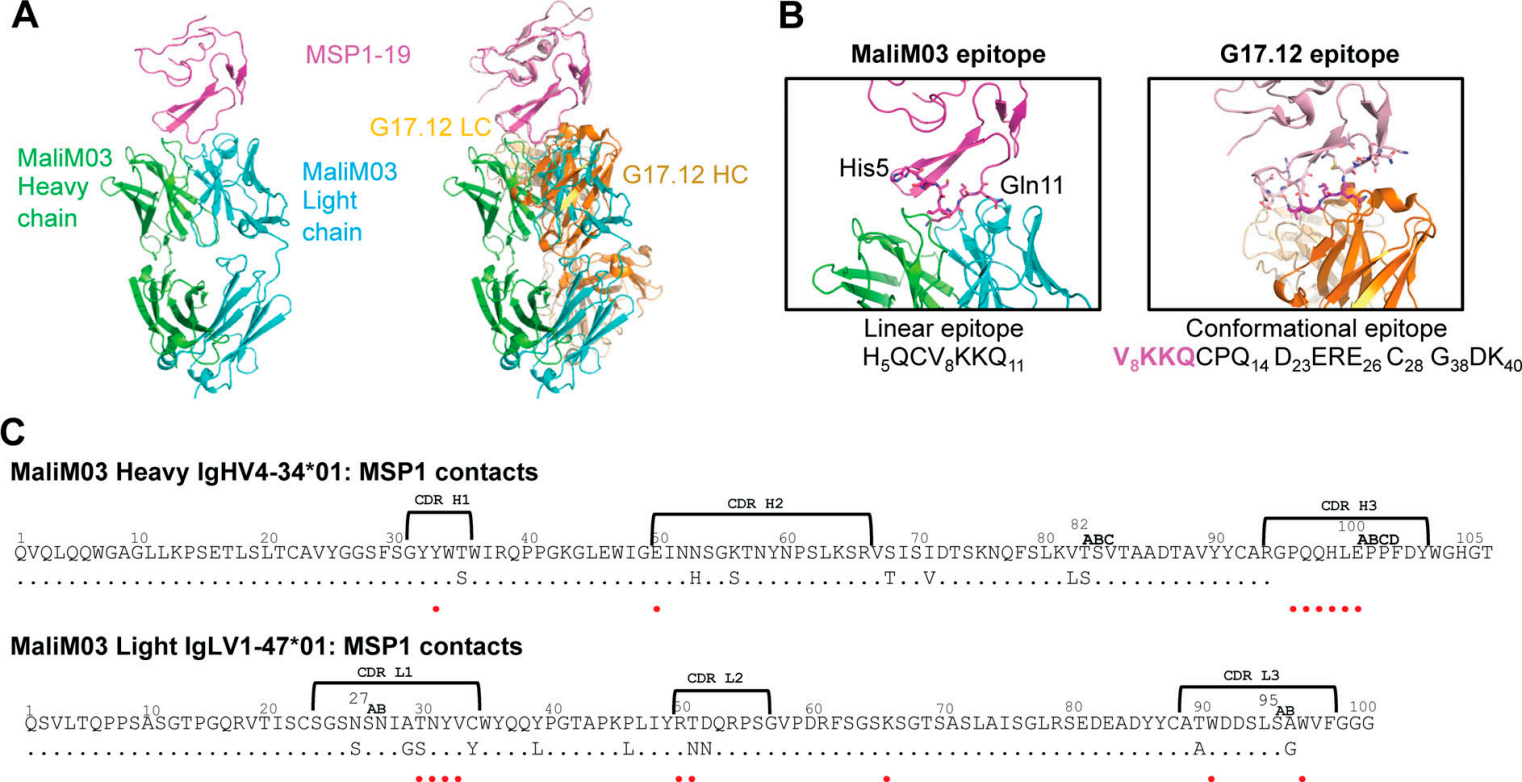
**Structure of MSP1 with MaliM03 Fab reveals a linear epitope at the N terminus of MSP1 with four residues in common with the conformational epitope of the monoclonal antibody G17.2.** **(A)** Crystal structure of the MaliM03 Fab fragment in complex with MSP1-19 (left), shown beside the previously published structure of G17.12 with MSP1. **(B)** A zoomed-in view of epitope binding for MaliM03 and G17.2. **(C)** Sequence of MaliM03 Fab using Kabat numbering and alignment with germline gene. Residues that are within 5 Å of MSP1 are indicated with red circles.

We continued our characterization of MaliM03. To visualize its epitope, we expressed it as an antigen-binding fragment (Fab), purified it in complex with MSP1-19 by size-exclusion chromatography, and set up sitting-drop crystallization trials. We obtained crystals that diffracted x rays to 3.0 Å (Fig. 4 A and Table S2). The solved structure indicates that 97% of the buried surface area of MSP1-19 upon MaliM03 binding involves a linear epitope at the N terminus of MSP1 (HQCVKKQ, corresponding to amino acids 5–11; Table S3). Interestingly, this epitope overlaps by four amino acids (VKKQ) with a previously described conformational epitope bound by the monoclonal antibody G17.12, raised by immunization of BALB/c mice with insect cells expressing recombinant MSP1 (Pizarro et al., 2003). The total buried surface area in the MaliM03–antigen complex is ∼1,042 Å^2^ (467 Å^2^ contributed from the MaliM03 Fab and ∼575 Å^2^ from MSP1), compared with ∼1,330 Å^2^ for the G17.12–antigen complex (∼670 Å^2^ contributed from the G17.12 Fab and ∼670 Å^2^ from MSP1). All MaliM03 complementarity-determining regions interacted with MSP1, whereas there were no interactions from the CDRL2 in the G17.12 Fab–bound complex (Pizarro et al., 2003). Although MaliM03 was derived from an IgM^+^ MBC, it exhibits evidence for somatic hypermutation of both the heavy and light chain components. In the light chain, 16 nucleotides are mutated from the germline V allele, resulting in 10 amino acid substitutions, two of which participate directly in epitope binding (Fig. 4 C and Table S3). The heavy chain also displays substantial hypermutation, with 18 mutated nucleotides resulting in seven amino acid changes, but none of these is directly involved in antigen binding.

**Figure 5.**
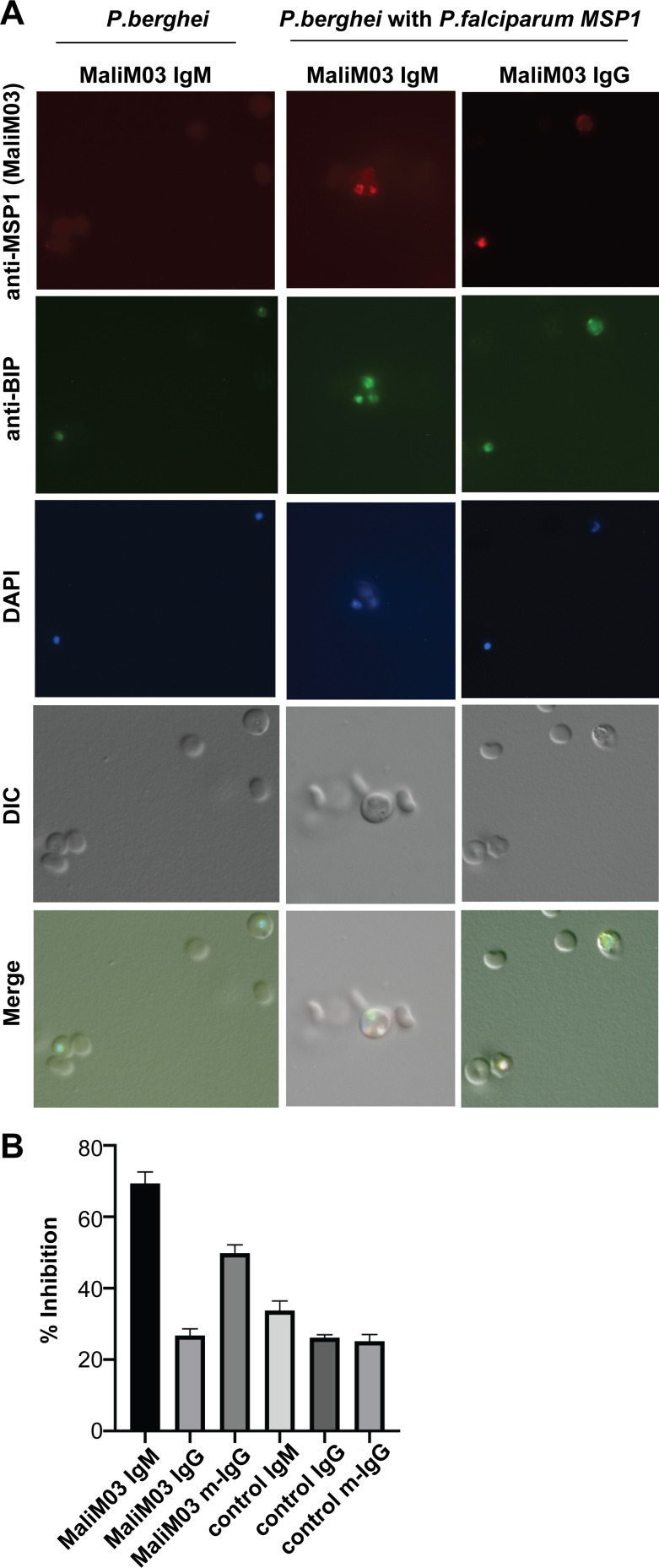
**Multimeric antibodies to MSP1 inhibit growth of *P. falciparum* better than the clonally identical monomer.** **(A)** Immunofluorescence assay showing binding of MaliM03 IgM or IgG to RBCs infected with *P. berghei* expressing MSP1 from *P. falciparum*. Wild-type *P. berghei* (which lacks the *P. falciparum* antigen) was used as a negative control. Parasites were colabeled with anti–*P. falciparum*–BIP to mark parasite endoplasmic reticulum and 6′-diamidino-2-phenylindole (DAPI) to stain nuclei. DIC, differential interference contrast. **(B)** Protective efficacy of MaliM03 IgM, IgG, and m-IgG was measured in a growth inhibition assay. IgG, IgM, and m-IgG forms of an irrelevant antibody were used as negative controls. Results shown are the mean + SEM pooled from two independent experiments.

We next used immunofluorescence assays to test whether MaliM03 could bind its antigen on intact *Plasmodium* parasites, a requirement for protective function. Both MaliM03 IgM and IgG molecules bound to transgenic *Plasmodium berghei* schizonts expressing *P. falciparum* MSP1 (Fig. 5 A; de Koning-Ward et al., 2003). In contrast, neither antibody bound to wild-type *P. berghei* parasites lacking the *P. falciparum* antigen, confirming specificity. The strength of IgM versus IgG binding cannot be compared directly in this assay due to the use of different secondary reagents to visualize bound antibodies.

A recent report showed that serum from human subjects with natural *Plasmodium* exposure contained anti-merozoite IgM and IgG, and inhibited merozoite invasion of RBCs in a complement-dependent manner (Boyle et al., 2019). On the other hand, the previously described MSP1-specific antibody G17.12 was capable of binding intact merozoites but did not inhibit their invasion into red blood cells (Pizarro et al., 2003). We tested MaliM03 IgM and IgG molecules in a growth inhibition assay for their ability to block parasite invasion. When merozoites were incubated with RBCs in the presence of MaliM03 IgM, their ability to invade new host cells was sharply restricted, demonstrating the protective efficacy of this multimeric antibody. In contrast, an equimolar concentration of the monomeric MaliM03 IgG molecule did not inhibit parasite growth any better than a control antibody specific for an irrelevant antigen (Fig. 5 B). Forced multimerization of the MaliM03 IgG molecule resulted in significantly better growth inhibition relative to the monomeric antibody, whereas multimerization of the control antibody did not enhance function. Thus, through multimerization, the MaliM03 antibody achieves strong parasite binding and potently protects RBCs from invasion.

## Discussion

Beginning with isolation of BCR sequences from single human MBCs and ending with antibody-dependent growth restriction of live parasites, our results demonstrate the efficacy of our pipeline for the discovery and expression of affinity-matured IgM molecules with extraordinarily strong antigen-binding properties. In addition, by engineering and characterizing clonally identical monomeric and m-IgG molecules alongside the original IgM, we show that multimerization of a moderate-affinity antibody can confer superior ability to bind antigen and restrict growth of parasites. Our results upend the traditional view of IgM as a low-affinity molecule that relies on multimerization to even approach the binding capacity of IgG. Instead, they support the concept that affinity-matured human IgM molecules, derived from CD21^+^CD27^+^IgM^+^ MBCs that have experienced repeated natural exposure to *Plasmodium,* not only may have decent affinity as monomers, but also may achieve exponentially better antigen-binding capacity compared with their IgG counterparts due to their high avidity.

The observation that the monomeric MaliM03 IgG antibody fails to block parasite invasion (Fig. 5 B) despite its ability to bind the parasite (Fig. 5 A) suggests that parasite binding by antibody is not sufficient for growth restriction, consistent with previous findings (Pizarro et al., 2003; Alanine et al., 2019). We suggest that the protective efficacy of MaliM03 IgM may depend in part upon a property of the multimer that is distinct from binding capacity, such as steric hindrance due to the large size of the complex or unique access to epitopes. Alternatively, restriction of parasite growth may require a binding strength that the IgM achieves through high-avidity interactions, whereas the affinity of the IgG monomer may not be sufficient to achieve the protective effect. These two possibilities are not mutually exclusive and may both contribute to the superior protective capabilities of multimeric MaliM03 IgM and m-IgG. Different inhibitory antibodies are known to restrict parasite growth in different ways, from neutralizing merozoites completely to slowing their rate of invasion into RBCs (Alanine et al., 2019). Although our growth inhibition experiments were conducted in the absence of immune effectors such as complement and phagocytes, the ability of multimerized constant regions to engage these additional effector functions might enhance their protective capacity in vivo. Altogether, these results underline the importance of multimeric antibodies in optimal humoral responses and suggest that IgM^+^ MBCs are important not only to maintain the plasticity of the MBC compartment but also to generate high-avidity antibodies with potent capacity to restrict pathogen growth during the anamnestic response. Our study also raises the possibility that high-affinity IgG antibodies might be engineered as multimers to confer avid binding and superior protection for the host.

Recent studies have found that IgM antibodies directed toward an immunodominant liver-stage antigen, circumsporozoite protein, can fix complement, lyse sporozoites, and block sporozoite traversal and invasion of hepatocytes (Zenklusen et al., 2018; Murugan et al., 2018; Behet et al., 2018). However, one study on human subjects immunized with RTS,S, the world’s first licensed malaria vaccine, found that levels of anti-sporozoite IgM correlated inversely with protection, suggesting a possible need to investigate other vaccine targets (Kurtovic et al., 2020). We propose that MaliM03, which targets a blood-stage antigen, is an excellent candidate for further investigation into the therapeutic potential of IgM molecules and will complement previous work targeting the liver-stage antigen.

Of note, we have identified a linear epitope within the conserved blood-stage antigen MSP1 that could perhaps be targeted to effectively inhibit growth of *P. falciparum*. Since vaccines directed at linear epitopes are straightforward to produce and are often more stable than those directed at conformational epitopes, which require proteins to remain intact in their native states (Oscherwitz, 2016), this knowledge may be informative for future vaccine design. In addition, our initial sequencing experiments identified a number of AMA1- and MSP1-specific antibodies other than MaliM03 that await further characterization, including MaliA01 and several more IgM antibodies. Finally, we suggest more generally that vaccine strategies, which until now have focused almost exclusively on production of IgG, may be improved by also considering elicitation of IgM^+^ MBCs.

## Materials and methods

### Study design and statistical analysis

Our research objective was to test the efficacy of somatically hypermutated, multimeric IgM molecules in binding and restricting *Plasmodium* parasites. To accomplish this, we isolated BCR sequences from IgM^+^ and IgG^+^ MBCs specific for *P. falciparum* antigens, expressed them as both IgM and IgG antibodies, and characterized their binding and functional properties. Sample sizes, number of replicates, and statistical tests used are included in each figure legend. Statistics were calculated using Prism 7 or 8 software.

### Human samples and flow-cytometric sorting

Deidentified human PBMC samples (*n* = 9) were from a previously described cohort of Malian subjects, age 10–15 yr, with a history of frequent *P. falciparum* exposure (Tran et al., 2013). The Ethics Committee of the Faculty of Medicine, Pharmacy and Dentistry at the University of Sciences, Techniques, and Technology of Bamako, Mali, and the Institutional Review Board of the National Institute of Allergy and Infectious Diseases, National Institutes of Health, approved the Mali cohort study (ClinicalTrials.gov identifier: NCT01322581). Written, informed consent was obtained from the parents or guardians of participating children. The MSP1 (p19 fragment) and AMA1 tetramers and labeling protocol have been described (Krishnamurty et al., 2016). Briefly, PBMCs were incubated with fluorophore-conjugated tetramer and a decoy reagent (Taylor et al., 2012), then with magnetic beads conjugated to an anti-fluorophore antibody. Labeled cells were enriched over a magnetic column and labeled with antibodies for surface markers (BD, Biolegend, or Invitrogen; Table S4). Single tetramer-labeled CD21^+^CD27^+^ cells were sorted into 96-well plates on an Aria (BD).

### BCR sequencing and antibody cloning

cDNA was extracted from single cells using a Maxima First Strand cDNA Synthesis Kit for RT quantitative PCR (Thermo Fisher Scientific), and BCR-specific transcripts were amplified in separate nested PCRs using DreamTaq Green PCR Master Mix 2X (Thermo Fisher Scientific). Amplicons were visualized on gels, and positive results were purified by incubating 5 μl of sample with 1 μl of FastAP (Thermo Fisher Scientific) and 0.5 μl Exonuclease I (Thermo Fisher Scientific) at 37°C for 30 min followed by 20 min at 85°C. Purified samples were sequenced in forward and reverse directions using a 3730 XL DNA Analyzer (Applied Biosystems). Results were trimmed using Geneious 9.1.3 (http://www.geneious.com/) at 0.01 error probability, and consensus sequences were generated for each chain using de novo assembly. Processed sequences were submitted to IMGT V-Quest (Giudicelli et al., 2011) for alignment. V and J genes were annotated from output tables, and somatic hypermutation frequency was determined by subtracting the IMGT V-region homology percentage from 100. Comparisons of mutation percentages between cell types for each chain was done using Graphpad Prism (Graphpad Software). Primers corresponding to the V and J genes were used with round one PCR product to generate amplicons used for cloning into expression vectors. Detailed methods and primers used have been previously described (Tiller et al., 2008).

To construct an m-IgG, the human IgM tail piece (PTLYNVSLVMSDTAGTCY) with 5′ and 3′ overlap regions for cloning was ordered as an ultramer from IDT. One additional mutation (L309C) and the IgM tail piece were introduced into an IgG heavy-chain construct (AbVec-hIgG1) by Gibson assembly.

### Antibody expression and purification

Protein expression was performed by transient transfection of 293T cells using polyethylenimine. IgM antibodies were expressed without the J chain; we observed no difference in binding capacity between preparations made with and without the J chain. For all experiments except the ELISA antigen-binding screen with IgM molecules (see below), cell culture supernatant was collected 6 d after transfection and purified using a HiTrap Protein G HP antibody purification column for IgG antibodies (GE Healthcare) and a POROS CaptureSelect IgM Affinity Matrix column (Thermo Fisher Scientific) for IgM. The final protein was buffer-exchanged into 1× PBS, filter-sterilized and stored at −80°C before use. SDS-PAGE analysis (NuPAGE 4–12% Bis-Tris Gel; Invitrogen) was performed to verify the formation and purity of the antibodies.

### ELISA

High-binding 96-well plates (Costar) were coated with recombinant *P. falciparum* MSP1, AMA1, or OVA (Sigma-Aldrich) as a negative control (each 5 µg/ml in PBS). For most experiments, after washing, purified recombinant antibodies were added at a maximum concentration of 5–50 nM, with three to five 10-fold serial dilutions, and incubated at room temperature for 2 h or overnight at 4°C. For the IgM ELISA shown in Fig. 2 C, ELISAs were performed using crude supernatants from transfected 293T cell cultures, with serial dilutions and incubations as above. Total IgM ELISAs were performed on supernatants to confirm antibody expression. Plates were washed, and HRP-conjugated anti-human IgG1 or anti-human IgM (1:1,000; Southern Biotech) was added for 1 h at room temperature. After a final wash, plates were developed with 3,3′,5,5′-tetramethylbenzidine substrate solution (Invitrogen), the reaction was stopped with 1 M HCl, and absorbance was measured at 450 nm.

### Negative-stain electron microscopy

To prepare the samples, IgG and IgM molecules were diluted to 0.01 mg/ml and 0.02 mg/ml, respectively, in 20 mM Tris-HCl, pH 7.9. Diluted samples were applied to glow-discharged carbon-coated grids and stained with 0.7% uranyl formate. Electron microscopy was performed on a Tecnai G2 Spirit (FEI Co.) operating at 120 kV, with images acquired at 67,000× magnification using a US4000 4K × 4K charge-coupled device camera (Gatan, Inc.).

### BLI

For measurements of IgM and IgG1 binding, antibodies were diluted in binding buffer (10 mM Hepes, pH 7.4, 150 mM NaCl, 3 mM EDTA, 0.05% surfactant P20, and 0.5% nonfat dry milk) and analyzed on an Octet RED96 System (ForteBio) with shaking at 1,000 rpm. Streptavidin-coated biosensors were incubated in wells containing biotinylated MSP1 p19 or AMA1 (25 nM) in binding buffer for 3–5 min for immobilization. A baseline measurement was made in buffer alone. Then, binding kinetics were monitored by dipping the biosensors in wells containing defined concentrations of the designed antibody (association), then dipping sensors back into baseline wells (dissociation). Data were processed with the instrument’s integrated software. Titrations were done in triplicate. Kinetic constants were determined from the mathematical fit of a 1:1 binding model (Berger et al., 2016).

Antigenicity assays to measure binding affinity without avidity effects were performed by diluting MaliA01 and MaliM03 IgG1 antibodies to 10 and 50 µg/ml, respectively, in Kinetics Buffer (1× Hepes-EP^+^ [Pall Forté Bio], 0.05% nonfat milk, and 0.02% sodium azide). AMA1 and MSP1 p19 were diluted to 50 nM and 3,000 nM in Kinetics Buffer and serially diluted twofold and threefold for final concentrations of 0.78 nM and 4.1 nM, respectively. Reagents were applied to a black 96-well Greiner Bio-one microplate at 200 µl per well as described below. Individual IgGs were immobilized onto ProteinA (ProA) biosensors per the manufacturer’s instructions (Forté Bio) except using the following sensor incubation times: ProA biosensors were hydrated in Kinetics Buffer for 10 min, and were then equilibrated in Kinetics Buffer for 60 s. The ProA tips were loaded with diluted IgGs for 150 s and washed with Kinetics Buffer for 60 s. The association step was performed by dipping the ProA biosensors with immobilized antibody into diluted AMA1 or MSP1 for 200 s. Then, dissociation was measured by inserting the biosensors back into Kinetics Buffer for 200 s. The data were baseline-subtracted and the plots fitted using the Pall Forté Bio/Sartorius analysis software (version 12.0).

### Protein expression and purification for crystallization

The MaliM03 IgG antibody was expressed by transfecting heavy and light chain plasmids into human embryonic kidney 293 Epstein–Barr virus nuclear antigen-1 cells (293 EBNA cells; catalog no. R620-07; Thermo Fisher Scientific) using 293 Free transfection reagent (Novagen). The IgG protein was purified from the supernatant using ProA resin (Goldbio) through a gravity flow column. The resin was washed with Protein Buffer (5 mM Hepes, pH 7.5, and 150 mM NaCl), and the IgG was eluted with Pierce IgG elution buffer (Thermo Fisher Scientific). The eluted IgG was buffer-exchanged into Protein Buffer and cleaved by Endoproteinase LysC (1 μg for 10 mg of protein; Roche) overnight at 37°C to obtain Fab. The *P. falciparum* MSP1 (p19) protein was cloned into the pTT3 vector and transfected in 500 ml 293 EBNA cells as described above. 6–7 d after transfection, filtered supernatant was incubated with nickel-nitrilotriacetic acid resin (Takara) overnight at 4°C and passed through a gravity flow column. Resin was washed with Protein Buffer containing 20 mM imidazole. The protein was eluted using Protein Buffer with 300 mM imidazole. Both Fab and MSP1 proteins were further purified on a size exclusion chromatography HiLoad 16/600 Superdex 200 column (GE) using Protein Buffer. The purified MaliM03 Fab and MSP1 proteins were mixed at a molar ratio of 1:1.5 and incubated at room temperature for 2–3 h. The complex was passed through the Hi Load 16/60 Superdex 200 column in Protein Buffer. The fractions corresponding to MaliM03Fab and MSP1 complex were pooled and concentrated to 15 mg/ml.

### Crystallization

The purified MaliM03Fab-MSP1 complex was screened for crystallization using an NT8 (Formulatrix)–dispensing robot. Screening was done with Additive screen (Hampton Research), Wizard Precipitant Synergy block no. 2 (Rigaku), and Crystal Screen HT (Hampton Research) in 96-well crystallization plates (MRC plates; Hampton Research) using the sitting drop vapor diffusion method. The protein drop was mixed with reservoir solution in a 1:1 ratio, equilibrated against 40 µl of reservoir solution, and incubated at 20°C. Good morphology crystals of MaliM03 Fab and MSP1 complex were obtained in a screening condition of 20% wt/vol polyethylene glycol (PEG) 8K, 40% vol/vol PEG 554 400, 0.5 M NaCl, and 0.1 M acetate buffer, pH 5.5 (Wizard Precipitant Synergy block no. 2; Rigaku). The screening condition was expanded using the hanging drop vapor diffusion method to obtain diffraction quality crystals. The crystals were cryoprotected in 15% ethylene glycol in mother liquor and flash-cooled in liquid nitrogen. Data were collected at the Advanced Light Source (ALS 5.0.2). The crystals diffracted to 3.0 Å resolution. The collected data were processed using HKL2000 (Otwinowski and Minor, 1997) and imported using Aimless in CCP4 (Collaborative Computational Project, Number 4, 1994). The structures were solved by molecular replacement using Phaser in CCP4 and Protein Data Bank accession no. 1OB1 as the search model (Pizarro et al., 2003). Structure building and refinement were performed in Phenix (Adams et al., 2010) and Crystallographic Object-Oriented Toolkit (COOT; Emsley and Cowtan, 2004), respectively. However, the electron density for residues 65–76 of MSP1 was not of good quality; therefore, those residues were not modeled. This crystal structure has been deposited in the Protein Data Bank (accession no. 6XQW).

### Immunofluorescence assay

Transgenic *P. berghei* ANKA parasites expressing *P. falciparum* MSP1 in place of the endogenous allele (de Koning-Ward et al., 2003) were grown in C57Bl/6 mice (Jackson Laboratories) housed under specific pathogen–free conditions. All animal work was performed with the approval of the University of Washington Institutional Care and Use Committee in accordance with the guidelines of the National Institutes of Health Office of Laboratory Animal Welfare. When parasitemia reached ∼5%, blood was harvested from euthanized animals by cardiac puncture, and red blood cells were washed and fixed in 4% paraformaldehyde with 0.0075% glutaraldehyde. Fixed cells were permeabilized with 10% Triton X-100 in PBS, blocked with BSA and incubated with MaliM03 IgM or IgG antibody along with a rabbit polyclonal anti-BIP antibody (kindly provided by Ashley Vaughan, Seattle Children’s Research Institute, Seattle, WA), followed by anti-rabbit antibody and anti-human IgM or IgG antibody conjugated to af488 and af594, respectively (Invitrogen). Finally, nuclei were stained with 6′-diamidino-2-phenylindole. PBS washes were performed between each step. Stained samples were applied to a slide and coverslipped using VectaShield. Images were obtained on a Nikon 90i microscope at 100× magnification.

### Growth inhibition assay

Antibodies were dialyzed against RPMI 1640 (KD Medical) and concentrated to 6.1 µM. For performing the growth inhibition assay, the antibodies were incubated at a final concentration of 3 µM with infected RBCs (*P. falciparum* 3D7 strain, 0.3% parasitemia at 1% hematocrit) in a final volume of 40 μl for 40 h at 37°C. A biochemical measurement using a *P. falciparum–*specific lactate dehydrogenase assay was used to determine parasitemia as described previously (Malkin et al., 2005).

### Data and materials availability

Sequences for all BCRs successfully amplified are in Table S1. Heavy and light chain sequences for MaliM03 and MaliA01 are also available in GenBank (accession nos. MW365929–MW365932). Coordinates and structure factors for PfMSP1 p19 in complex with MaliM03 Fab have been deposited in the Protein Data Bank (accession no. 6XQW).

### Online supplemental material

Fig. S1 shows the somatic hypermutation of BCR V segments in each study donor. Table S1 shows all BCR sequences and alignment data obtained from sorted MBCs. Table S2 shows the data collection and refinement statistics for x-ray crystallography of MSP1 with MaliM03. Table S3 describes interactions between MSP1 and MaliM03 in detail. Table S4 lists the antibodies used for flow cytometry.

## Supplementary Material

Table S1 shows BCR sequence and alignment data from sorted MBCs.

Table S2 shows data collection and refinement statistics for crystal structure.

Table S3 shows detailed interactions between PfMSP1-19 and MaliM03 Fab (from Pisa web server, http://www.ebi.ac.uk).

Table S4 shows anti-human antibodies used to label *Plasmodium*-specific B cells for flow-cytometric sorting.
