# Supplementary material for: Multimeric antibodies from antigen-specific human IgM+ memory B cells restrict Plasmodium parasites
Source: J Exp Med. 2021 Mar 4;218(4):e20200942. doi: 10.1084/jem.20200942 (PMC7938364; doi:10.1084/jem.20200942)
Supplement: Table S2 — shows data collection and refinement statistics for crystal structure. [file JEM_20200942_TableS2.docx]

**Table S2.** Data collection and refinement statistics for crystal structure

|  | **PfMSP1-19/MaliM03 Fab** |
| --- | --- |
| **Data collection** |  |
| Space group | C121 |
| Cell dimensions |  |
| *a*, *b*, *c* (Å) | 119.74, 69.45, 65.83 |
| *α*, *β*, *γ* (°) | 90, 108.24, 90 |
| Resolution (Å) | 50.00-3.00 (3.05–3.00)* |
| *R*_sym_ or *R*_merge_ | 0.103 (0.437)* |
| *I*/s*I* | 10.7 (2.0)* |
| Completeness (%) | 99.9(99.2)* |
| Redundancy | 3.3 (3.1)* |
| CC_1/2_ | 0.990 (0.971)* |
|  |  |
| **Refinement** |  |
| Resolution (Å) | 47.02–2.99 (3.29–2.99)* |
| No. reflections | 10,453 (2,448)* |
| *R*_work_/*R*_free_ | 25.16/30.92 (31.76/42.96)* |
| Protein atoms |  |
| Chain H  Chain L  Chain E | 1,584  1,545  507 |
| B-factors (Å^2^) | 64.77 |
| Protein  Chain H  Chain L  Chain E | 58.98  61.27  93.55 |
| R.m.s. deviations |  |
| Bond lengths (Å) | 0.005 |
| Bond angles (º) | 0.66 |
| Ramachadran Favored % | 92.32 |
| Ramachadran Outliers % | 0.00 |
| MolProbity all-atoms clashscore | 10.27 |
|  |  |
| **PDB ID** | **6XQW** |

* Statistics for the highest-resolution shell are shown in parentheses.
