# Supplementary material for: Multimeric antibodies from antigen-specific human IgM+ memory B cells restrict Plasmodium parasites
Source: J Exp Med. 2021 Mar 4;218(4):e20200942. doi: 10.1084/jem.20200942 (PMC7938364; doi:10.1084/jem.20200942)
Supplement: Table S3 — shows detailed interactions between PfMSP1-19 and MaliM03 Fab (from Pisa web server, http://www.ebi.ac.uk). [file JEM_20200942_TableS3.docx]

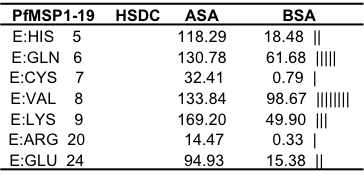

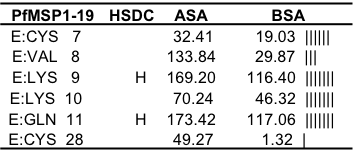

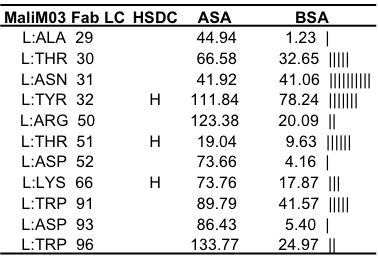

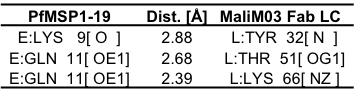

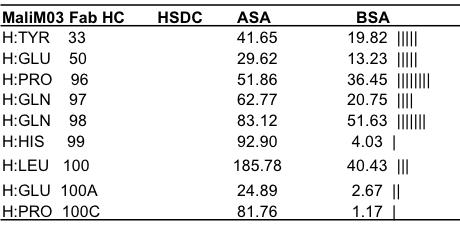


**Table S3. Detailed interactions between PfMSP1-19 and MaliM03 Fab (from Pisa web server, http://www.ebi.ac.uk)**

a. Detailed interactions of PfMSP1-19 and MaliM03 Fab Heavy Chain (HC)

b. Detailed interactions of PfMSP1-19 and MaliM03 Fab Light Chain (LC)

Hydrogen Bonds

**HSDC H**ydrogen/**D**isulphide bond, **S**alt bridge or **C**ovalent link; **ASA**  Accessible Surface Area, Å²;

**BSA**  Buried Surface Area, Å²  ||||   Buried area percentage, one bar per 10%
