# Supplementary material for: Multimeric antibodies from antigen-specific human IgM+ memory B cells restrict Plasmodium parasites
Source: J Exp Med. 2021 Mar 4;218(4):e20200942. doi: 10.1084/jem.20200942 (PMC7938364; doi:10.1084/jem.20200942)
Supplement: Table S4 — shows anti-human antibodies used to label Plasmodium-specific B cells for flow-cytometric sorting. [file JEM_20200942_TableS4.docx]

**Table S3. Anti-human antibodies used to label *Plasmodium*-specific B cells for flow cytometric sorting.**

| **Antibody Specificity** | **Clone** | **Color** |
| --- | --- | --- |
| CD19 | HIB19 | Alexa Fluor 700 |
| CD20 | 2H7 | PerCpCy5.5 |
| CD3 | UCHT1 | BV711 |
| CD14 | MOP9 | BV711 |
| CD16 | 3G8 | BV711 |
| IgM | MHM-88 | BV510 |
| IgD | IA6-2 | PE-Cy7 |
| IgG | G18-145 | BV786 |
| CD21 | HB5 | APC |
| CD27 | M-T271 | BV421 |
